# Programmable Optical Synaptic Linking of Neuromorphic Photonic-Electronic RTD Spiking Circuits

**DOI:** 10.1021/acsphotonics.4c01199

**Published:** 2024-10-01

**Authors:** Matěj Hejda, Weikang Zhang, Qusay Raghib Ali Al-Taai, Ekaterina Malysheva, Dafydd Owen-Newns, José M. L. Figueiredo, Bruno Romeira, Joshua Robertson, Victor Dolores-Calzadilla, Edward Wasige, Antonio Hurtado

**Affiliations:** † Institute of Photonics, SUPA Dept of Physics, University of Strathclyde, Glasgow G11XQ, United Kingdom; ‡ Hewlett-Packard Laboratories, Hewlett-Packard Enterprise, Machelen 1831, Belgium; ¶ High Frequency Electronics Group, University of Glasgow, Glasgow G128QQ, United Kingdom; § Eindhoven Hendrik Casimir Institute, Eindhoven University of Technology, Eindhoven 5600MB, The Netherlands; ∥ Centra-Ciências and Departamento de Física, Faculdade de Ciências, Universidade de Lisboa, Lisboa 1649004, Portugal; ⊥ INL − International Iberian Nanotechnology Laboratory, Ultrafast Bio- and Nanophotonics Group, Braga 4715330, Portugal

**Keywords:** optical synaptic linking, spike weighting, neuromorphic, optoelectronic, resonant tunneling diodes, RTD

## Abstract

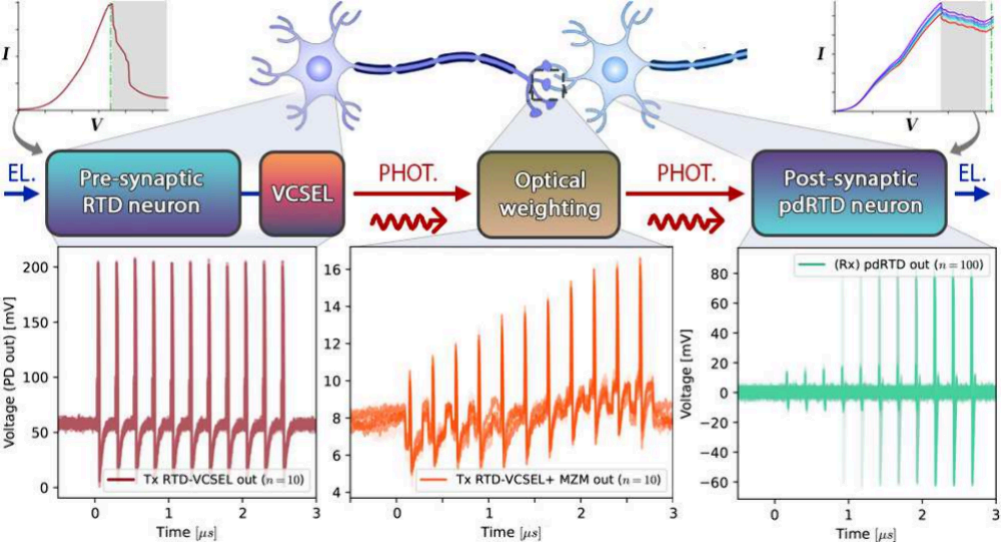

Interconnectivity between functional building blocks (such as neurons and synapses) represents a fundamental functionality for realizing neuromorphic systems. However, in the domain of neuromorphic photonics, synaptic interlinking and cascadability of spiking optical artificial neurons remains challenging and mostly unexplored in experiments. In this work, we report an optical synaptic link between optoelectronic spiking artificial neurons based upon resonant tunneling diodes (RTDs) that allows for cascadable spike propagation. First, deterministic spiking is triggered using multimodal (electrical and optical) inputs in RTD-based spiking artificial neurons, which are optoelectronic (OE) circuits incorporating either micron-scale RTDs or photosensitive nanopillar-based RTDs. Second, feedforward linking with dynamical weighting of optical spiking signals between pre- and postsynaptic RTD artificial neurons is demonstrated, including cascaded spike activation. By dynamically weighting the amplitude of optical spikes, it is shown how the cascaded spike activation probability in the postsynaptic RTD node directly follows the amplitude of the weighted optical spikes. This work therefore provides the first experimental demonstration of programmable synaptic optical link and spike cascading between multiple fast and efficient RTD OE spiking artificial neurons, therefore providing a key functionality for photonic-electronic spiking neural networks and light-enabled neuromorphic hardware.

## Introduction

**Figure 1:**
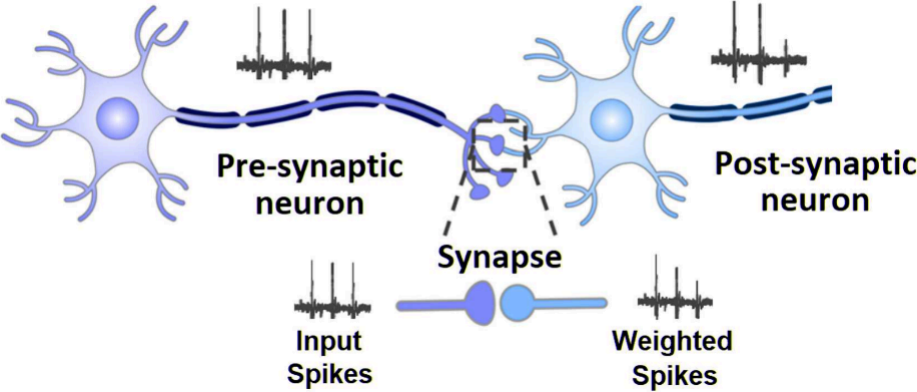
Schematic diagram of a synaptic link between two biological neurons. Spikes fired by the presynaptic neuron are propagated through its axon toward a second postsynaptic neuron. The strength of the connection is mediated through a synapse, which we typically model as weighting in artificial neural networks. The postsynaptic neuron fires in response to synapse-adjusted signals from upstream presynaptic neuron(s).

With the recent emergence of powerful artificial intelligence (AI) approaches such as large language models (LLMs), AI-powered tools are now poised to become truly ubiquitous. However, there are some notable challenges related to the current state of AI computing. Running state-of-the-art large AI models such as LLMs requires massive amounts of computing resources, including specialized hardware such as graphical or tensor processing units (GPUs, TPUs^1^), among others. Furthermore, the energy consumption^2^ and carbon footprint of running LLMs^3^ is extensive. The goal of overcoming these challenges represents the main driving force behind the pursuit for novel, unconventional computing hardware. Neuromorphic engineering is one of the major research directions for next-generation computing substrates.^4^ Inspired by the workings of biological brains and relying on concepts such as spike-based signaling (Figure 1) and spiking neural networks, high parallelism, non-von Neumann or in-memory computing, neuromorphic chips represent a promising solution particularly in more energy constrained and edge-focused use-cases. Furthermore, over the past decade, neuromorphic photonics^5^ has emerged as a rapidly growing research field bringing together the increasing demands for AI acceleration and neuro-inspired computing with new optical technologies such as photonic integration. In particular, neuron-like optical spiking and spike-based information processing have been demonstrated in a wide range of photonic devices and architectures, with nonexhaustive list of examples including multisection integrated lasers,^6−8^ various approaches based on integrated microring resonators (MRRs)^9−11^ and optoelectronic approaches with cointegrated excitable CMOS circuits^12^ or superconducting circuits,^13^ among others.

Resonant tunnelling diodes (RTDs) represent a class of active semiconductor devices capable of achieving ultrahigh (THz-range) bandwidth,^14^ rendering them as promising devices for applications in communications.^15^ RTD circuits can be considered as a case of Liénard oscillators,^16^ and therefore exhibit complex nonlinear dynamics that include excitable spiking and bursting^17^ which allows them to function as spiking artificial neurons for neuromorphic circuits. These will be further referred to as RTD nodes. Furthermore, RTDs can also exhibit photodetection (this has been shown down to single photon levels^18^), which allows for activation of excitable spiking dynamics directly in response to optical input signals.^19^ Thanks to this functionality and their nonlinear dynamical responses, RTDs are actively investigated for use as artificial spiking neurons in high-speed, efficient neuromorphic photonic-electronic systems.^20^ However, experimental demonstrations of more complex interconnected layouts (employing more than a single spiking RTD node) remain unexplored.

For operation of an interconnected RTD system with optical inputs/outputs, the electronic-to-optical (E/O) conversion of the spiking RF signals produced by the RTDs is performed using a directly modulated vertical cavity surface emitting laser (VCSEL) coupled through a bias-tee network.^21^ VCSELs have a strong track record in the field of neuromorphic photonics,^22^ with previous reports demonstrating injection locked VCSELs as spiking encoders with precise timing and rate-based encoding.^23^ Some of the applications in the neuromorphic domain include all-optical spike weighting units when operated as optical amplifiers^24^ as well as photonic reservoir computers^25^ or extreme learning machines.^26^

## Methods

**Figure 2:**
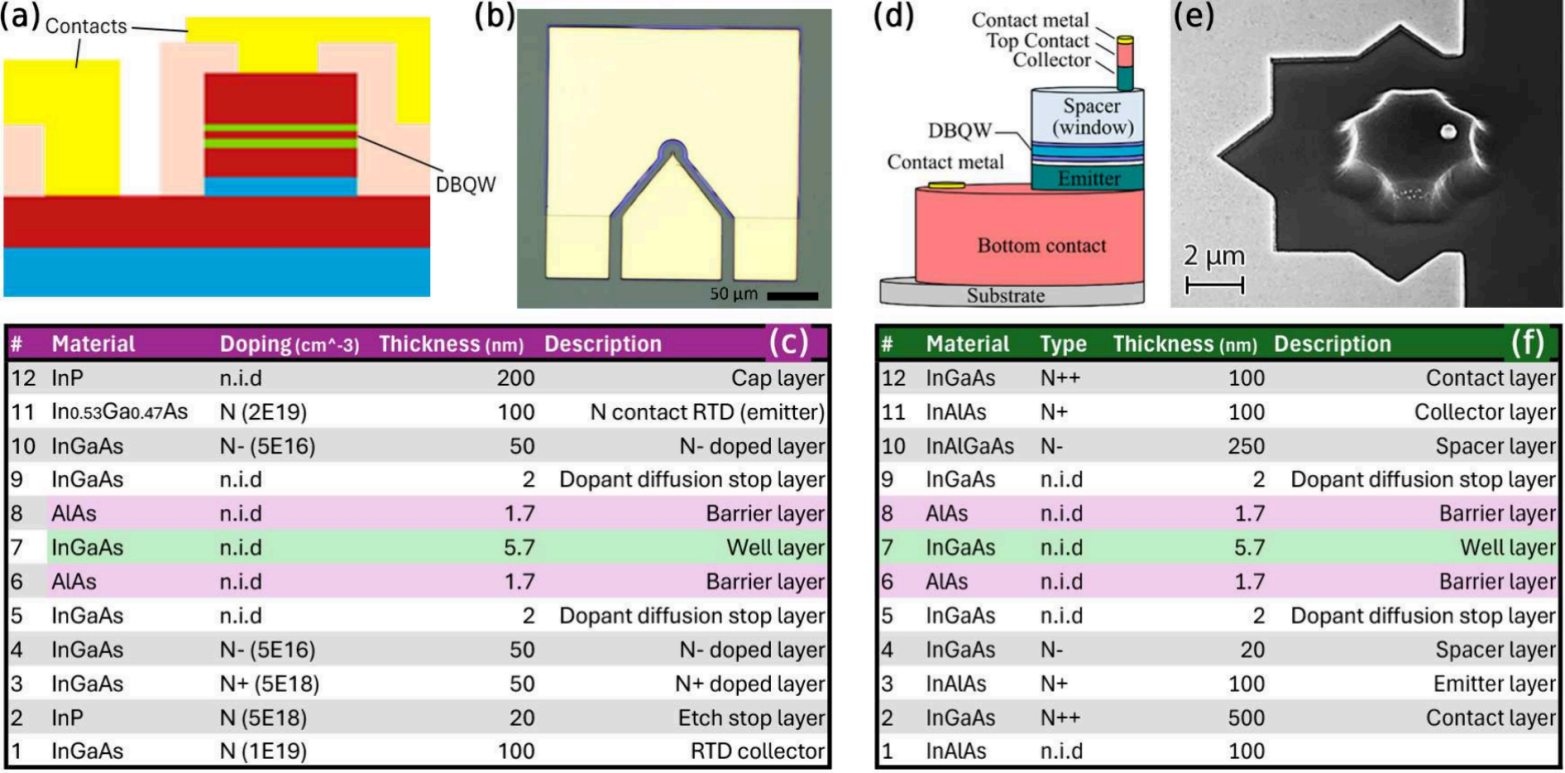
(a) Cross-section of the μRTD. (b) Microscope image of the whole μRTD device. (c) Epitaxial layer stack of the μRTD. (d) Schematic of the npRTD device design. (e) SEM image of the npRTD, with the individual nanopillar appearing as a small, white-colored structure at top. (e) Epitaxial layer stack of the npRTD.

This work studies functional neuromorphic photonic arrangements based upon a pair of excitable RTD optoelectronic artificial neurons. These are referred to as presynaptic (transmitter, Tx) and postsynaptic (receiver, Rx) neurons, respectively. Two different types of RTD devices are used: *(a)* an electrically triggered RTD with 3 μm mesa radius which will be further referred to as **μRTD** (micron-scale RTD); *(b)* photosensitive, nanopillar-based RTDs^19^ with an optical window, which permits these devices to elicit electrical spikes in response to multimodal (electrical or infrared optical) inputs. Therefore, devices of this type will be further referred to as **npRTDs** (nanopillar photosensitive RTDs). The epitaxial layer stacks, cross sections and micrographs of both devices are shown in Figure 2. Further details regarding the npRTD devices used in this work are available in.^19^

**Figure 3:**
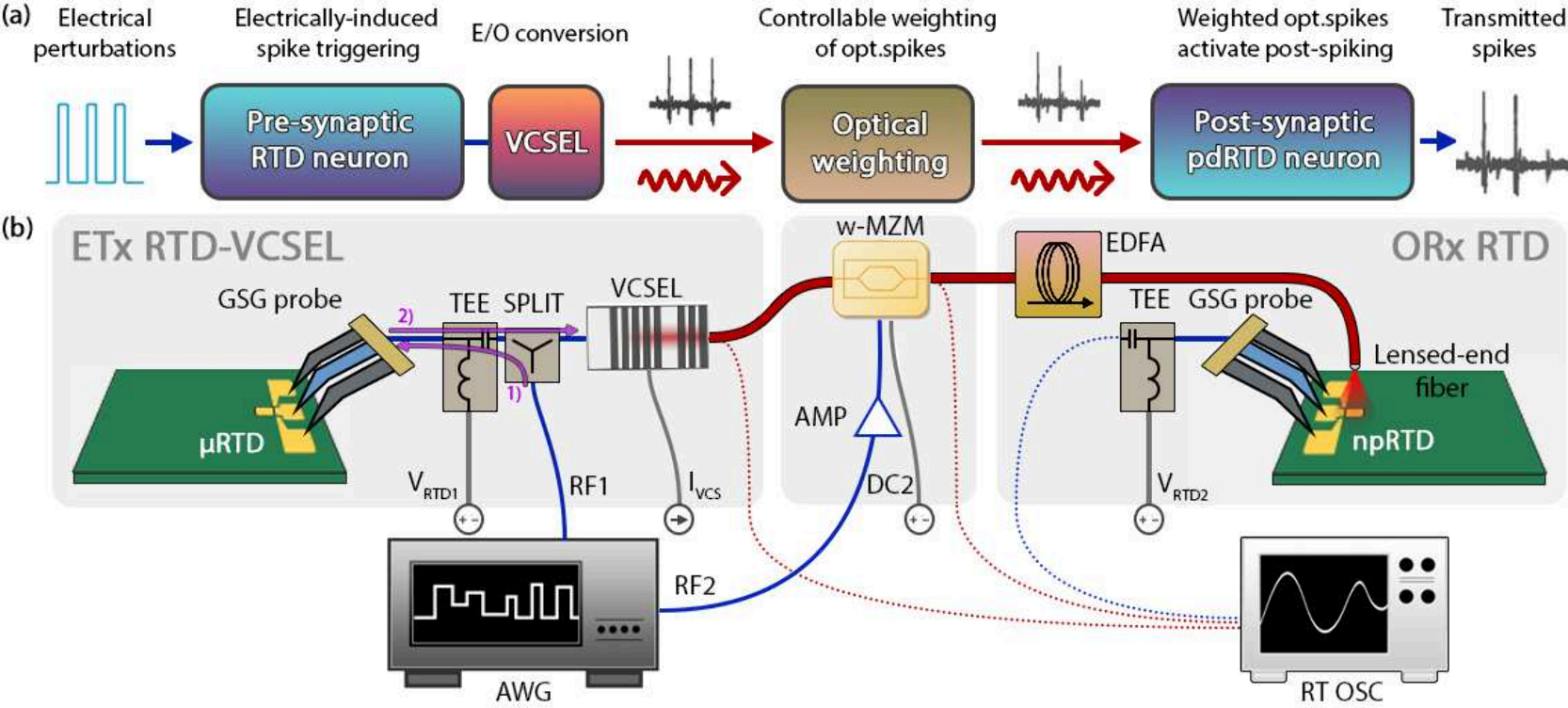
(a) Schematic diagram and (b) experimental setup of the synaptic optical link between two RTD spiking artificial neurons, including a presynaptic μRTD-VCSEL node and a postsynaptic npRTD node. The demonstration includes a dynamical spike weighting stage realized using a Mach–Zehnder modulator (MZM). An RF signal from an AWG elicits electrical spiking events in the presynaptic neuron. The electrical spiking output is converted to the optical domain by a 1550 nm VCSEL and propagated to the postsynaptic npRTD neuron through the synaptic link. There, the optical spikes are individually dynamically weighted at the MZM following a patterned weight mask also generated by the AWG. Finally, the individually weighted optical spikes are directly injected into the postsynaptic npRTD neuron (via a lensed-end optical fiber), which responds with a final sequence of spikes. AMP: wideband RF amplifier, TEE: bias-tee, SPLIT: resistive RF power splitter. *Optical connections (fibers) are shown in red; RF connection are shown in blue; DC connections are shown in gray*.

First, in the *Electrical Tx-Optical Rx (ETx-ORx)* synaptic architecture, the presynaptic node is based upon a spiking μRTD triggered with an electrical input, while the postsynaptic node is a single npRTD with a 700 nm nanopillar triggered using a direct optical input to the device. Second, in the *Optical Tx-Optical Rx (OTx-ORx)* synaptic architecture, both pre- and postsynaptic nodes are realized using the same type of npRTD devices with a 500 nm nanopillar. This allows us to elicit spikes in both the pre- and postsynaptic devices by directly using optical pulses of sufficient (superthreshold) power.^19^ All the output timetraces are collected using a 16 GHz real-time oscilloscope (RT OSC), including a 9 GHz amplified photodetector (Thorlabs PDA8GS) where optical signals are recorded (shown as dotted red signal paths in Figure 3, Figure 5 below).

### ETx-ORx (Electrical Tx-Optical Rx) Synaptic Architecture

Figure 3(a) shows a schematic signal flow diagram of the ETx-ORx experiment, with corresponding experimental setup description shown in Figure 3(b). The presynaptic (Tx) μRTD neuron in this ETx-ORx synaptic link architecture was realized using a μRTD device directly coupled to a low-energy VCSEL operating at standard telecom wavelength λ_*VCSEL*_ = 1550 nm, therefore realizing an OE artificial spiking neuron.^21^ The resulting optical spikes from the presynaptic μRTD-VCSEL neuron are transmitted via a single-mode (SM) fiber-optic link which includes a dynamic optical weighting stage via a Mach–Zehnder Modulator (MZM). For maximizing optical power, an optional fiber polarization controller (PC) was used prior to the MZM to match the polarization to the modulator. The weighted optical spikes are further amplified using an erbium doped fiber amplifier (EDFA) and directly injected into the postsynaptic (Rx) npRTD neuron using a lensed-end fiber. In response to these weighted optical spikes, the postsynaptic node may fire electrical spikes. The experiment includes a 2-channel 12 GSa/s arbitrary waveform generator (AWG), where the first channel (RF1) provides a series of electrical square-shaped pulses (stimuli; Figure 7(a)) that directly trigger spiking in the presynaptic μRTD, and the second channel (RF2) modulates the MZM, therefore allowing for dynamical optical intensity weighting.

**Figure 4:**
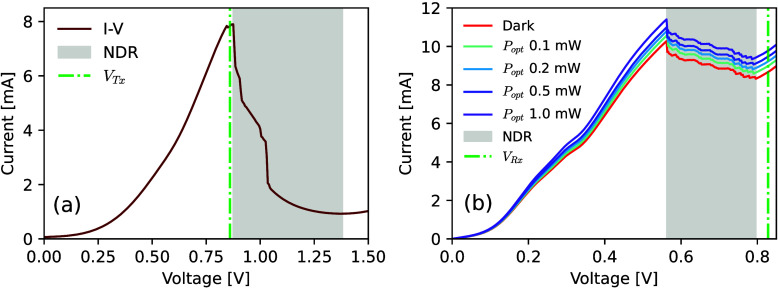
Experimentally measured *I*-*V* curves of the RTDs acting as pre- and postsynaptic artificial neurons in the ETx-ORx synaptic link. (a) Measured *I*-*V* characteristic of the μRTD device used to realize the presynaptic μRTD-VCSEL neuron. This shows a high-contract NDR region ranging from approximately 875 mV to 1.38 V. (b) Measured *I*-*V* curve of the npRTD implementing the postsynaptic neuron. The IV curve was measured under dark conditions (red line) and under illumination of 1550 nm CW laser light with varying optical power. In this case, the NDR can be observed between 563 mV and 796 mV. The npRTD is operated in the reverse bias mode.

The presynaptic μRTD is forward-biased at *V*_*Tx*_ = 860 mV, in the first positive differential resistance (PDR) region and in a close vicinity of the peak point of the *I*-*V* characteristic. Figure 4(a); shows the bias with a green dash-dotted line, negative differential resistance (NDR) region between approximately 875 mV to 1.38 V is highlighted in gray. This device permits the generation of excitable electrical spikes in response to small electrical input perturbations.^21^ The peak-to-valley current ratio (PVCR) of this device is high (approximately 8.5), resulting in spikes with high spike amplitude. The VCSEL was coupled to the μRTD^27^ for E/O conversion of the spiking signals via direct modulation. It has a lasing threshold of *I*_*T*_ = 1.95 mA and is DC biased at *I*_*VCS*_ = 3.23 mA, which corresponds to 210 μW CW-equivalent optical power at VCSEL output. For the postsynaptic npRTD, the device has a 700 nm mesa diameter and a circular optical window with 9 μm diameter. The measured *I*-*V* characteristic of this npRTD is shown in Figure 4(b), revealing an NDR region between 563 mV and 796 mV. Here, the *I*-*V* was acquired both under dark conditions and under continuous wave (CW), infrared (λ = 1550 nm) illumination through its optical window (up to 1 mW). This reveals how external light inputs influence the *I*-*V*, therefore allowing external optical perturbations (pulses) to directly elicit excitable electrical responses in the npRTD, here triggered in response to (weighted) optical spikes from the presynaptic μRTD-VCSEL node. The npRTD was reverse-biased at *V*_*Rx*_ = 828.3 mV, in the second PDR and in close vicinity of the valley point of the *I*-*V* characteristic (Figure 4(b), shown with a green dash-dotted line).

### OTx-ORx (Optical Tx-Optical Rx) Synaptic Architecture

**Figure 5:**
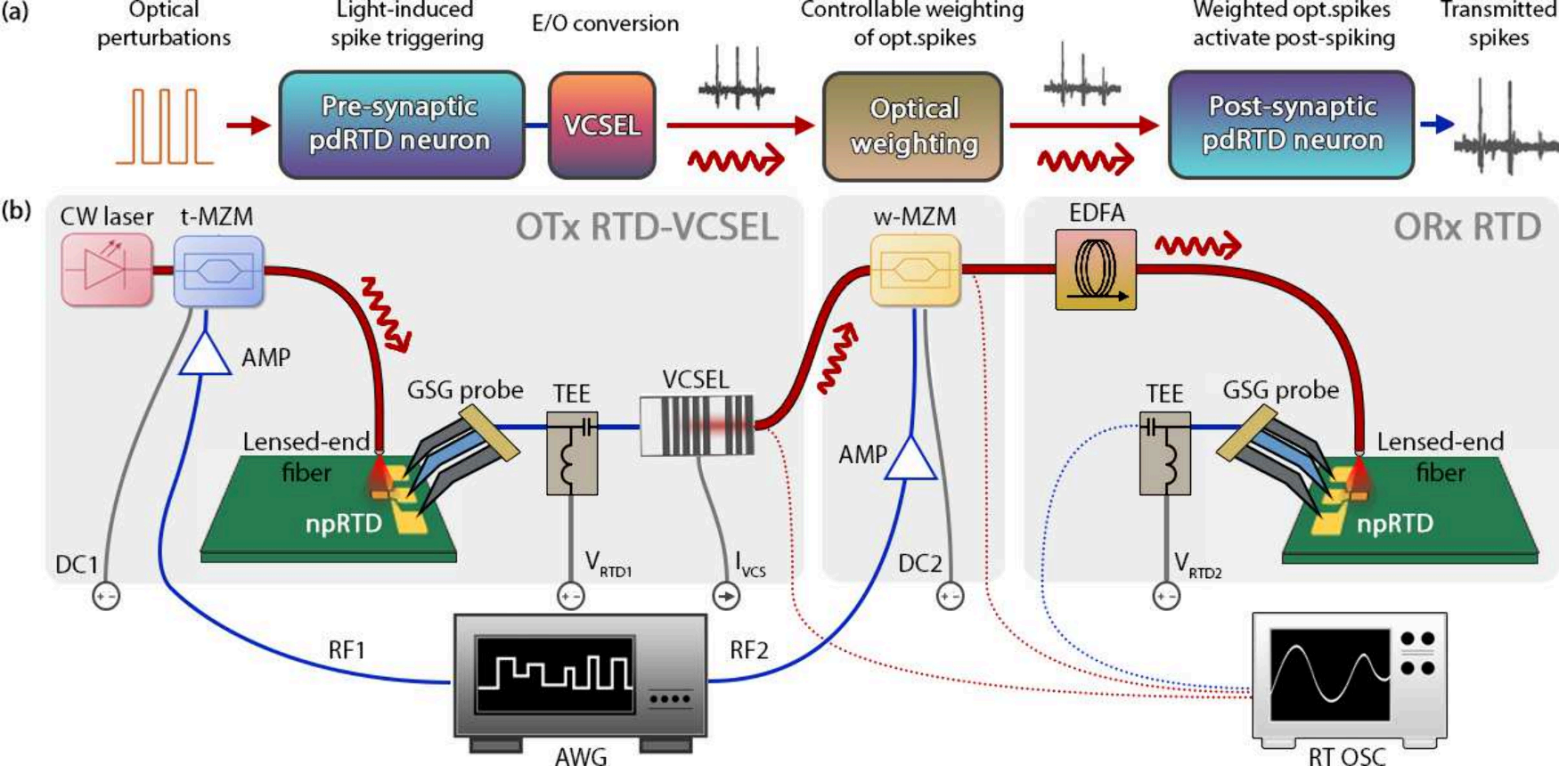
(a) Schematic diagram and (b) experimental setup of the synaptic optical link between two RTD spiking artificial neurons, including an optically triggered, presynaptic npRTD-VCSEL node and a postsynaptic npRTD node. The demonstration includes a dynamical spike weighting stage realized using a Mach–Zehnder modulator (MZM). An RF signal from an AWG is used to modulate CW light from a 1550 nm tunable CW laser and provide the optical stimuli for spike activation in the presynaptic μRTD neuron. The spiking output is converted to the optical domain by a 1550 nm VCSEL and propagated to the postsynaptic npRTD neuron through the optical synaptic link. There, the optical spikes are individually dynamically weighted at the MZM following a patterned weighting mask also generated by the AWG. Finally, the individually weighted optical spikes are directly injected into the postsynaptic npRTD neuron (via a lensed-end optical fiber), which responds with a final sequence of electrical spikes. AMP: wideband RF amplifier, TEE: bias-tee, SPLIT: resistive RF power splitter. *Optical connections (fibers) are shown in red; RF connection are shown in blue; DC connections are shown in gray.*

Figure 5(a) shows a schematic signal flow diagram of the OTx-ORx architecture (Figure 5(a)) and the experimental setup used to implement it (Figure 5(b)). Optical signal with fast (ns-rate) perturbations for activating the presynaptic spiking node is realized using a CW tunable laser (TL, at 1550 nm) that is modulated using an MZM, referred to as t-MZM (triggering-MZM). Both the presynaptic (Tx) and postsynaptic (Rx) nodes are realized using photosensitive nanopillar-based npRTD devices^19^ with 500 nm mesa diameter (smaller in comparison to the ETx-ORx case) and 5 μm × 5 μm square-shaped optical window.

The presynaptic node also incorporates a 1550 nm VCSEL coupled through a bias-tee to the npRTD circuit for E/O conversion via direct current modulation of the VCSEL. The presynaptic npRTD-VCSEL and postsynaptic npRTD artificial neurons are unidirectionally interconnected through a SM optical fiber link. As is in the case of the ETx-ORx architecture, this link incorporates a single weighting Mach–Zehnder modulator (w-MZM) for dynamical optical weighting of the presynaptic optical spikes, and an EDFA to ensure sufficient optical power level prior to optically coupling the signals to the npRTD postsynaptic spiking neuron (via lensed-end optical fiber) . An optical power level of 600 μW was set post-EDFA, at the input of the lensed-end fiber coupled to the postsynaptic npRTD. The lensed-end optical fiber injects weighted optical spikes directly into the npRTD device, which then fires optically induced excitable electrical spikes in response. An AWG provides two RF modulation signals, one for the perturbations in t-MZM (RF1) and one for controlling the w-MZM (RF2).

**Figure 6:**
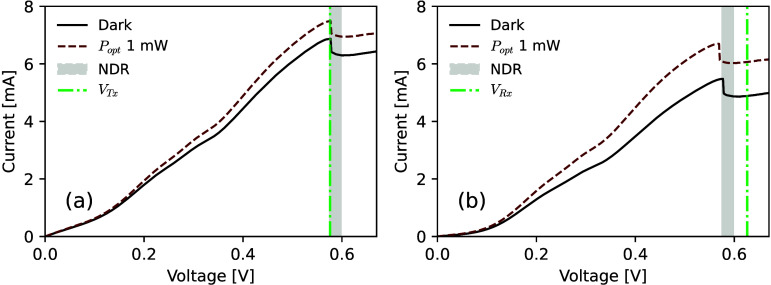
Experimentally measured *I*-*V* curves of the nanopillar RTDs operating as pre- and postsynaptic artificial neurons in the OTx-ORx synaptic link. (a) *I*-*V* characteristic of the npRTD used to realize the presynaptic npRTD-VCSEL neuron. (b) *I*-*V* curve of the npRTD implementing the postsynaptic neuron. Both devices were reverse biased and measured under both dark conditions (black solid line) and under illumination (1 mW CW optical signal at 1550 nm; red dashed line). Both devices also exhibits an NDR region in a similar range of applied voltage biases, from approximately 575 mV to 598 V.

The *I*-*V* curves of the presynaptic npRTD are shown in Figure 6(a), and the *I*-*V*s of the postsynaptic npRTD are shown in Figure 6(b). All of the *I*-*V* curves in Figure 6 exhibit the key property of NDR (gray shaded area). In both cases, measurement were performed under no illumination (darkness; solid black line) and under 1 mW of 1550 nm CW laser light input, with the signal being injected to the device through a lensed-end fiber (dashed red line). The npRTD devices exhibit a shift in their measured *I*-*V* curves when subject to infrared light illumination; hence permitting activation of excitable spikes upon the arrival of optical stimuli.^19^ The presynaptic npRTD device was biased with a voltage *V*_*Tx*_ = 581 mV (Figure 6(a), green dash-dotted line), just below the peak point of the device’s *I*-*V* characteristic. The postsynaptic npRTD was biased with a voltage *V*_*Rx*_ = 626 mV (Figure 6(b), green dash-dotted line), which is an operation point adjacent to the valley point of the device’s *I*-*V* characteristic. The coupled VCSEL has a lasing threshold of *I*_*T*_ = 1.95 mA and is DC biased at *I*_*VCS*_ = 2.3 mA, corresponding to CW-equivalent power of 210 μW.

## Results

### Experimental ETx-ORx Results

**Figure 7:**
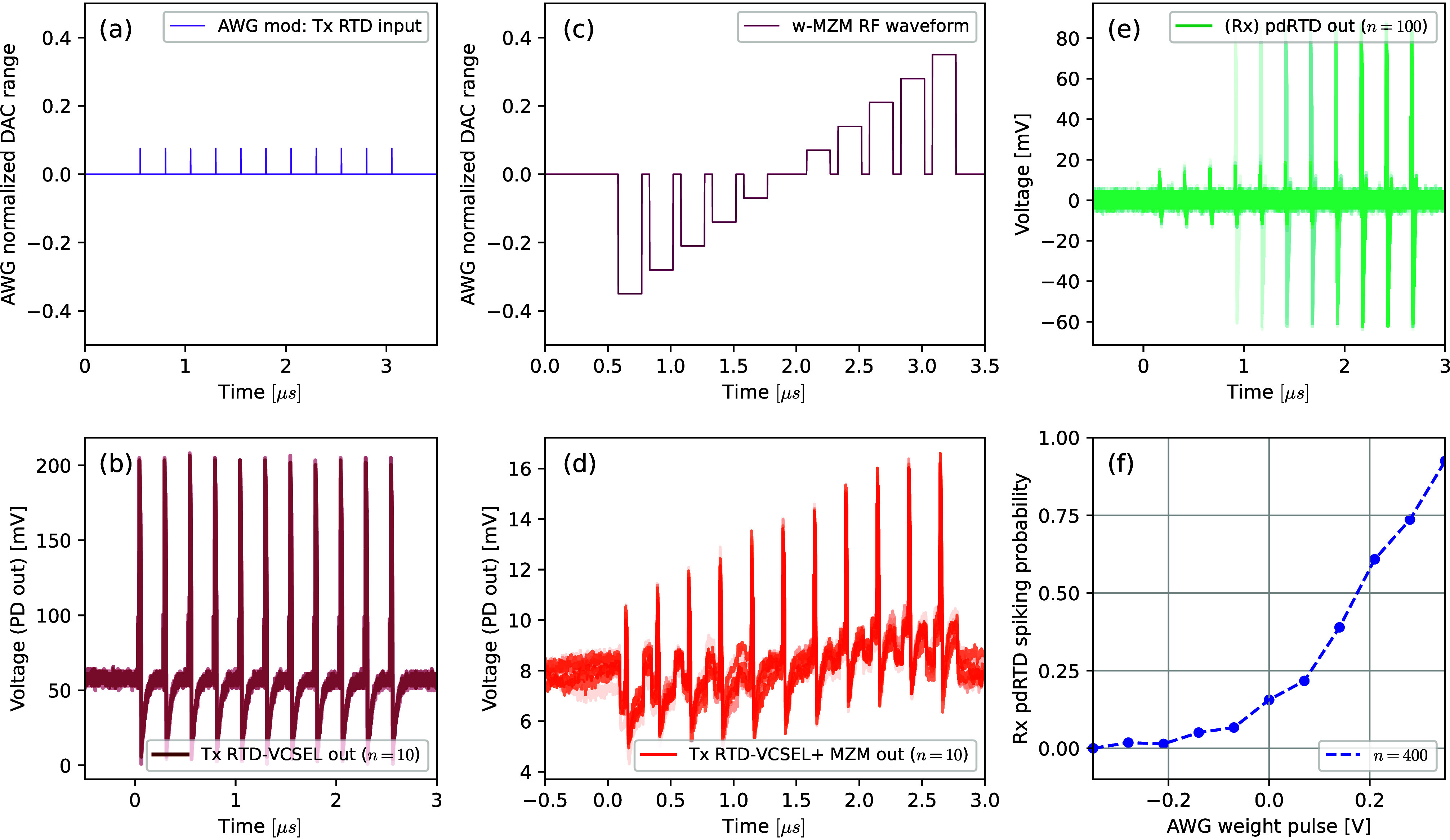
Experimental results demonstrating the ETx-ORx neuromorphic synaptic link with incorporated optical spike weighting functionality. (a) Timetrace of the RF modulation signal, providing the perturbations that elicit deterministic spikes in the presynaptic μRTD-VCSEL node. (b) Spiking optical output from the presynaptic μRTD-VCSEL node. (c) RF waveform used to perform the dynamic optical weighting functionality via the w-MZM. (d) Weighted optical spikes, prior to optical amplification and injection into the postsynaptic npRTD. The spikes have enhanced/suppressed intensities depending on the weighting factor applied via the w-MZM. (e) Overlay of *n* = 100 spiking responses of the postsynaptic npRTD artificial neuron in response to the weighted optical spikes from the presynaptic neuron. (f) Normalized spike firing probability of the postsynaptic npRTD artificial neuron in response to the weighted optical spikes from the presynaptic μRTD-VCSEL node (calculated from *n* = 400 RT OSC readouts).

Figure 7 plots the experimentally measured time traces illustrating the operation of the ETx-ORx synaptic architecture. The electrical spiking regimes in the presynaptic μRTD-VCSEL artificial neuron were triggered using a RF modulation signal (RF1 from the AWG, Figure 7(a)), consisting of fast (500 ps-long) square-shaped, positive 75 mV pulses with a temporal separation of 250 ns (measured between rising edges). Trigger pulse polarity was positive to match the forward-biased, peak-point operated μRTD device. This approach allows for highly reliable, deterministically elicited excitable spiking in RTDs^21^ which is E/O converted in the coupled VCSEL. The optical output time trace of this presynaptic μRTD-VCSEL artificial neuron is shown in Figure 7(b). In the interlink, the weighting MZM (w-MZM) was set at a fixed operational bias in the semilinear region of its transfer function and modulated with an RF signal from the AWG (RF2) consisting of gradually varying square-shaped pulses with return-to-zero (RZ) characteristic. This weighting RF “staircase” waveform is shown in Figure 7(c) and consists of 187.5 ns long varying amplitude pulses interleaved with RZ sections of 62.5 ns (75% duty cycle). Thanks to the VCSEL being biased with a current close to its lasing threshold, its CW power level is reduced. The dual polarity nature of the RF signal driving the w-MZM was used to better utilize the maximum peak-to-peak RF signal amplitude generated from the digital-to-analog converter (DAC) in the AWG. The weighted optical spikes after the w-MZM are shown in the time trace included in Figure 7(d), demonstrating the well-controlled, dynamical spike-synchronized optical weighting operation at ns-rates in the ETx-ORx neuromorphic synaptic optical link.

Finally, prior to entering the postsynaptic node, the weighted optical signal was optically amplified using an EDFA, targeting an average post-EDFA optical power of 480 μW. The input was injected to the postsynaptic npRTD using a lensed-end optical fiber vertically coupled to the exposed region (optical window) of the device. The readout was performed by directly measuring the electrical (RF) response of the postsynaptic npRTD artificial neuron. Figure 7(e) shows the measured output timetrace of the postsynaptic npRTD spiking neuron following the arrival of the weighted optical spikes. In total, *n* = 400 readouts were acquired using the RT OSC, and *n* = 100 are shown (overlaid) in Figure 7(e). These time traces confirm that weighted optical spikes from the presynaptic μRTD-VCSEL artificial neuron which have lower absolute amplitude directly correspond to lower spike activation probability in the postsynaptic npRTD neuron. Furthermore, the number of spike firing events triggered in the postsynaptic npRTD neuron was counted over all the recorded readouts, and plotted in the histogram included in Figure 7(f). This histogram validates the observation that optically induced spike firing probability in the postsynaptic npRTD is directly related to the dynamically adjusted weighting value applied on the optical spikes from the presynaptic μRTD-VCSEL neuron in the ETx-ORx neuromorphic link.

### Experimental OTx-ORx Results

**Figure 8:**
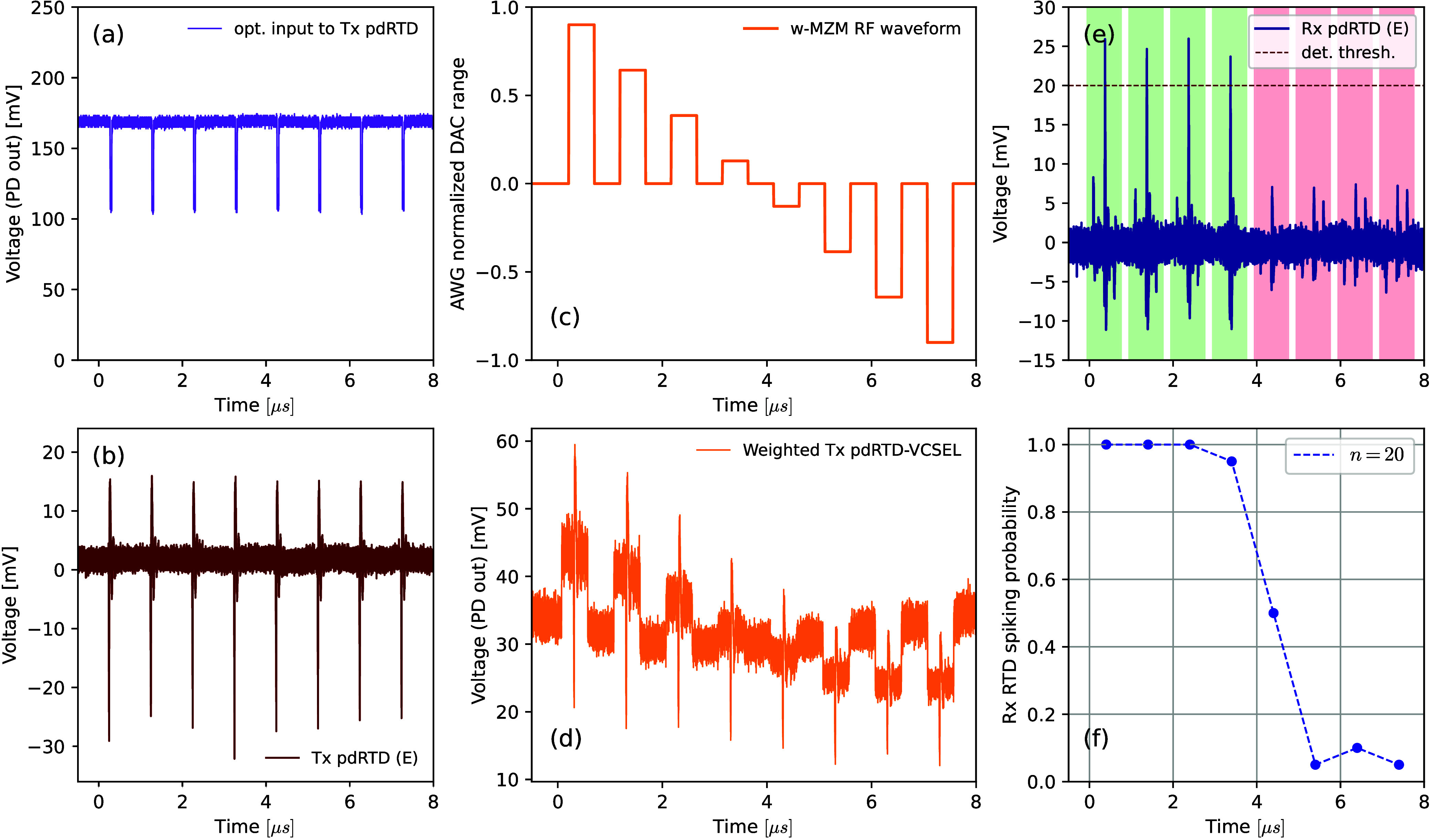
Experimental results demonstrating of the OTx-ORx neuromorphic synaptic link with the incorporated optical spike weighting functionality. (a) Timetrace of the optical input signal (from the TL, modulated with t-MZM) used to deterministically trigger spiking in the presynaptic npRTD-VCSEL node. (b) Spiking output from the presynaptic npRTD. (c) RF waveform used to perform the dynamic optical weighting functionality via the w-MZM. (d) Weighted optical spikes, prior to optical amplification and injection into the postsynaptic npRTD. The spikes have enhanced/suppressed intensities depending on the weighting factor applied via the w-MZM. (e) Spiking responses of the postsynaptic npRTD in response to the weighted optical spikes from the presynaptic neuron. (f) Normalized spike firing probability of the postsynaptic npRTD in response to the weighted optical spikes from the presynaptic npRTD node (calculated from *n* = 20 RT OSC readouts).

Figure 8 shows the experimental results of dynamic spike propagation and weighting in the OTx-ORx architecture. Figure 8(a) depicts the input timetrace with the modulated signal from the tunable laser carrying the optical amplitude perturbations, which are used to deterministically elicit spiking events in the presynaptic npRTD-VCSEL artificial neuron. These trigger perturbations (RF1 from AWG) are set as 10 ns-long square pulses with a repetition interval of 500 ns that are polarity matched (negative) to the operation regime of the presynaptic npRTD (reverse biased device operated at a peak-adjacent operation point of the *I*-*V* curve). These triggering perturbations elicit spikes from the presynaptic npRTD node, shown in the time trace in Figure 8(b). This spiking RF signal is converted to the optical domain by direct modulation of the 1550 nm VCSEL coupled to the npRTD. The optical spikes from the presynaptic npRTD-VCSEL node are then optically weighted by the w-MZM in the synaptic link. To achieve dynamical optical weighting of all individual optical spikes from the presynaptic node, the return-to-zero stepped RF signal (RF2 from AWG) shown in Figure 8(c) is applied to the w-MZM. The resulting optical signal containing the dynamically weighted spikes is shown in Figure 8(d). These are optically amplified with an EDFA and injected into the postsynaptic npRTD artificial neuron, which fires electrical excitable spike events in response. The electrical spiking output signal from the postsynaptic npRTD output was directly measured with the RT OSC, and is shown in Figure 8(e). The latter shows that only the first 4 incoming (weighted) optical spikes from the presynaptic neuron have sufficient amplitude to exceed the spike firing threshold of the postsynaptic npRTD neuron, therefore eliciting spikes at its output. The remaining weighted input optical spikes do not elicit a spiking response due to their reduced amplitude, yielding only a small photoresponse. The time trace shown in Figure 8(e) represents a mean time trace over *n* = 10 measurements. In addition, the probability of triggering a downstream spike in the postsynaptic neuron (over *n* = 20 repeated time trace acquisitions) is shown in Figure 8(f). These data confirm that the cascadable spike activation process is highly reproducible, with only a small degree of ambiguity existing right at the spike activation threshold where, for example, noise effects can directly influence the spike activation in the npRTD neuron.

## Discussion

In both the ETx-ORx and OTx-ORx cases demonstrated in this work, we utilize peak-point biased Tx nodes and valley-point biased Rx nodes. Spiking in the Tx RTD was in both cases deterministically activated with external perturbations (either electrical or optical). Typically, the polarities of both the trigger signal and the elicited spike are inverted between the peak and valley biasing operation points. Furthermore, spikes produced under the peak-point or valley-point biasing regimes can exhibit different temporal lengths or refractory period. Here, the ETx node is operated in a forward bias, and requires positive voltage pulses to elicit spiking responses. In the second case, the OTx node is operated under a reverse bias condition, and requires negative pulses (optical power drops) for activating spikes at the peak point. The OTx also accordingly produces downward (”negative”) spikes. Both the ORx nodes are reverse biased in the valley, and therefore produce upward (”positive”) spikes. In our experiments, we have observed that this combination of peak-Tx and valley-Rx biasing yielded the most reliable spike propagation. Therefore, if we theoretically extrapolate to a deep feed-forward spiking neural network (SNN) architecture, an alternating peak/valley biasing between subsequent layers represents a favorable setting. Since our synaptic link controls the amplitude of the spikes, our architecture is suitable for SNN implementations where weighting is performed via amplitude, similar to graded spikes used for example in Intel’s Loihi 2 chips.^28^

In Figure 6, we can observe a certain degree of variation between the OTx and ORx npRTD devices. Currently, these can primarily be attributed to process tolerances during the npRTD fabrication. In the excitable RTD circuit, the difference between the individual biasing voltage *V*_*Tx*_, *V*_*Rx*_ and the voltage of the device’s *I*-*V* extrema (peak or valley) determines the excitable threshold distance from the steady state. The closer an RTD is biased to its *I*-*V* peak (or valley), the lower is the required energy for incoming perturbations (optical or electrical) to trigger a spiking response. For an operation in an optoelectronic (OE) SNN, the spiking threshold distance from the steady state is likely to represent an important hyperparameter (playing a role very similar to bias in ANNs). Therefore, adjustable (or adaptive) biasing control of RTD circuits should also allow the OE SNN to adapt to these minor variations between the different *I*-*V* characteristics of individual RTDs.

Practically, our OE spiking approach represents an alternative to all-optical spiking lasers, including injection-locked VCSELs,^22^ VCSELs with saturable absorber (VCSEL-SA)^29^ and integrated multisection lasers with saturable absorber such as Fabry–Perot^30,31^ and DFB lasers.^32^ The advantage of our OE approach is primarily in higher degree of operational robustness, since the OE circuit is less sensitive to variations in characteristics of input optical signals (wavelength, polarization etc.). While all-optical approaches currently offer higher spike firing rates, further optimizations of the RTD OE circuit (and its parasitics) are likely to further improve the speed of the spiking dynamics, from current refractory period-limited maximum firing of ≈10 MHz^21^ toward the GHz region. To compare the power consumption levels of ETx-ORx and OTx-ORx nodes, we can approximate the *P* = *I* × *V* using the recorded *I*-*V* characteristics, and assuming the mean voltage is approximately equal to the bias applied to the RTD circuit. For the VCSEL in the Tx nodes, we can coarsely estimate the power draw from the measured output optical power in our experiments (210 μW) using the estimated wall plug efficiency (WPE) for this class of lasers (WPE ≈10%^33^). The power consumption is then estimated as *P* = *P*_*TxRTD*_ + *P*_*VCSEL*_ + *P*_*RxRTD*_. For ETx-OTx, this yields *P*_*EO*_ = 15.5 mW, while for OTx-ORx, this yields *P*_*OO*_ = 7.7 mW. Therefore, at the level of spiking nodes, the nanopillar-based RTDs draw 50% less power, further strengthening the case for use of npRTDs in the system. Furthermore, while the current implementation utilizes optical amplification via EDFAs to ∼0.5 mW optical power levels for more reliable operation, we have also observed devices where spiking could be elicited at ∼100 μW optical input power (measured prior to lensed-end fiber). Therefore, we believe that further optimizations of RTD device design (such as the epilayer stack) and optical coupling efficiency into the npRTDs may yield architectures that will allow reliable operation without the optical signal amplification stage.

## Conclusions

In summary, this work reports experimentally for the first time on neuromorphic photonic synaptic linking and cascadability between RTD-based OE spiking artificial neurons with <100 mV/sub-mW electrical/optical activations in a two neuron (presynaptic/transmitter, and postsynaptic/receiver) arrangement. We demonstrate that spikes from one RTD OE artificial neuron can be optically communicated to another RTD OE artificial neuron yielding controllable spike firing patterns in response. Moreover, we demonstrate this synaptic linking operation for electrical-optical (ETx-ORx) and optical–optical (OTx-ORx) architectures. First, the ETx-ORx architecture uses an electrical μRTD as the presynaptic excitable device, and a nanopillar photosensitive npRTD as a postsynaptic excitable device. The ETx-ORx demonstrates a well-defined dynamically adjustable optical spike weighting stage and optical cascadability of the weighted spikes. Second, the OTx-ORx link demonstrates the synaptic interlinking functionality between two optically triggered, low-power npRTD neurons with lower signal levels. Such nanoscale RTDs have previously been reported as promising devices for ultralow power, high-speed neuromorphic nanophotonics.^34^ In both the ETx-ORx and OTx-ORx cases, the presynaptic nodes include a coupled discrete 1550 nm VCSEL for E/O conversion, and a Mach–Zehnder modulator (w-MZM) included in the link for a high-speed dynamic optical spike weighting, permitting on-demand tuning of the individual amplitude of all the ns-rate optical spikes transferred in the link.

These two (ETx-ORx, OTx-ORx) synaptic architectures highlight the versatility of RTD-based neuromorphic spike-processing systems, allowing multimodal (photonic or electronic) data inputs and operation using spikes of different polarities (positive or negative) depending on the desired use case. We believe the current proof-of-concept based on discrete components and fiber optics offers a validation step toward practical optoelectronic spiking neural networks (OE SNNs). With next research steps aimed toward realizing interlinked, RTD-powered OE circuits in fully integrated photonic platforms,^35^ we envision RTDs as a key enabling technology for high-speed and efficient photonic-electronic neuromorphic hardware.
